# Cerebrospinal fluid leakage after cranial surgery in the pediatric population—a systematic review and meta-analysis

**DOI:** 10.1007/s00381-021-05036-8

**Published:** 2021-02-04

**Authors:** Emma M. H. Slot, Kirsten M. van Baarsen, Eelco W. Hoving, Nicolaas P. A. Zuithoff, Tristan P. C van Doormaal

**Affiliations:** 1 Department of Neurology and Neurosurgery, University Medical Center Utrecht, Utrecht, The Netherlands; 2 Department of Neuro-oncology, Princess Máxima Center for Pediatric Oncology, Utrecht, The Netherlands; 3 Julius Center for Health Sciences and Primary Care, University Medical Center Utrecht, Utrecht, The Netherlands; 4 Department of Neurosurgery, Clinical Neuroscience Center, University Hospital Zurich, Zurich, Switzerland

**Keywords:** Cerebrospinal fluid leakage, Craniotomy, Craniectomy, Posterior fossa surgery, Pediatrics

## Abstract

**Background:**

Cerebrospinal fluid (CSF) leakage is a common complication after neurosurgical intervention. It is associated with substantial morbidity and increased healthcare costs. The current systematic review and meta-analysis aim to quantify the incidence of cerebrospinal fluid leakage in the pediatric population and identify its risk factors.

**Methods:**

The authors followed the PRISMA guidelines. The Embase, PubMed, and Cochrane database were searched for studies reporting CSF leakage after intradural cranial surgery in patients up to 18 years old. Meta-analysis of incidences was performed using a generalized linear mixed model.

**Results:**

Twenty-six articles were included in this systematic review. Data were retrieved of 2929 patients who underwent a total of 3034 intradural cranial surgeries. Surprisingly, only four of the included articles reported their definition of CSF leakage. The overall CSF leakage rate was 4.4% (95% CI 2.6 to 7.3%). The odds of CSF leakage were significantly greater for craniectomy as opposed to craniotomy (OR 4.7, 95% CI 1.7 to 13.4) and infratentorial as opposed to supratentorial surgery (OR 5.9, 95% CI 1.7 to 20.6). The odds of CSF leakage were significantly lower for duraplasty use versus no duraplasty (OR 0.41 95% CI 0.2 to 0.9).

**Conclusion:**

The overall CSF leakage rate after intradural cranial surgery in the pediatric population is 4.4%. Risk factors are craniectomy and infratentorial surgery. Duraplasty use is negatively associated with CSF leak. We suggest defining a CSF leak as “leakage of CSF through the skin,” as an unambiguous definition is fundamental for future research.

**Supplementary Information:**

The online version contains supplementary material available at 10.1007/s00381-021-05036-8.

## Introduction

Cerebrospinal fluid (CSF) leakage is one of the most common complications after neurosurgical intervention. CSF leakage is associated with substantial morbidity and increased healthcare costs [10]. One study found an average cost difference of €17.412 for patients with postoperative CSF leakage compared to patients without CSF leakage [10]. CSF leakage may lead to the development of a pseudomeningocele (PMC), wound healing problems requiring surgical re-closure, surgical site infection, meningitis, and pneumocephalus. CSF leakage rates reported in pediatric studies range between 0 and 38% [6, 12, 17, 28, 33]. Definitions of CSF leakage vary in the existing body of literature.

The exact magnitude of the problem in children, however, is still unknown and may be larger than in adults for several reasons. First, almost half of all pediatric brain tumors resides in the posterior fossa, and posterior fossa surgeries are thought to be more prone to CSF leakage [10, 16, 31]. Second, intraventricular tumors are more common in the pediatric population [31]. Surgical opening of the ventricle may result in higher chance of postoperative CSF leakage [1]. A clear understanding of the incidence and risk factors of CSF leakage in the pediatric population is essential in the prevention of CSF leakage in children. The current systematic review and meta-analysis aim to address these issues.

## Methods

The authors followed the PRISMA guidelines[25] for this systematic review and meta-analysis.

### Search strategy and selection criteria

Embase, PubMed, and Cochrane databases were searched until August 31, 2020 for studies reporting CSF leakage and related complications after intradural cranial surgery in patients up to 18 years old. The following search terms were used: ““children” OR “child” OR “pediatric” OR “paediatric” OR newborn OR “adolescent” OR “infant”” AND “neurosurgery” OR “craniotomy” OR “craniectomy” OR “cranial surgery” OR “tumor resection” AND ““cerebrospinal fluid leakage” OR “CSF leakage”” OR ““pseudomeningocele” OR “incisional leakage” OR “wound leakage” OR “surgical site infection” OR “surgical wound infection” OR “meningitis”” and relevant Mesh/Emtree terms. A modified version of the filter used to search pediatric studies in PubMed is used [22] (see Appendix A–C for the full search strings). Studies written in other languages than English, Dutch, German, French, Italian, or Spanish were excluded. Studies written before 1966 were excluded, as those are not included in the PubMed database. Laboratory studies, animal studies, cadaveric studies, case reports, small case series (*N* < 10), and literature reviews were excluded. Furthermore, studies on transsphenoidal surgery, skull base reconstructions, burr hole surgery (i.e., drainage of chronic subdural hematoma, needle biopsy), and primary CSF diversion surgeries were excluded. Two authors (EMHS and KMvB) independently screened all records from the database search on title and abstract to identify relevant articles. All remaining full text articles were screened on their eligibility for inclusion. A consensus meeting was held to reach agreement on the included articles.

### Data extraction

The following patient specific data items were extracted as proportion or mean per study: age, gender, compromised immune status, previous chemotherapy or radiotherapy, presence of hydrocephalus preoperatively, and CSF diversion surgery (endoscopic third ventriculostomy (ETV)/external ventricle drain (EVD)/ventriculoperitoneal (VP) shunt). The following surgical items were collected as proportion per study: site of durotomy (infratentorial/supratentorial), craniotomy versus craniectomy, indication for surgery (i.e., tumor resection or Chiari decompression), ventricular opening (yes/no), use of sealant (yes/no), use of duraplasty (yes/no), and whether a “watertight” closure of the dura was attempted or not. The following outcome parameter was collected: proportion of patients with CSF leakage (based on the individual study’s definition).

Study quality was assessed according to the National Heart, Lung and Blood Institute of National Institutes of Health (NIH) quality assessment tool for case series studies [27]. Studies with more than 2 items with high risk for bias or unclear risk for bias were classified as poor quality. Studies with a maximum of 2 items with high risk for bias or unclear risk for bias were judged to be of fair quality. Studies with no items with high risk of bias and a maximum of 1 item with unclear risk of bias were deemed of good quality.

### Statistical analysis

A meta-analysis of the incidence of CSF leakage was performed using a generalized linear mixed model. Heterogeneity of the data across studies was determined using Higgins *I*^2^ [13].

The primary outcome measure in this study is the incidence of CSF leakage with 95% confidence interval (CI). Subgroup analyses were performed for the separate surgical indications Chiari decompression (with dural opening) and posterior fossa tumor surgery. Secondary outcome measures are the odds ratio (OR) for CSF leakage for craniotomy versus craniectomy, supratentorial versus infratentorial surgery, cases in which a duraplasty was used or not, and studies in which watertight closure was attempted in all cases or not. Finally, three sensitivity analyses were performed (1) for studies of high quality only, (2) for studies of > 50 patients only, and (3) including the study of Jiang et al (see Results section) [17].

All analyses were performed using SAS version 9.4 (SAS Institute Inc).

## Results

**Fig. 1:**
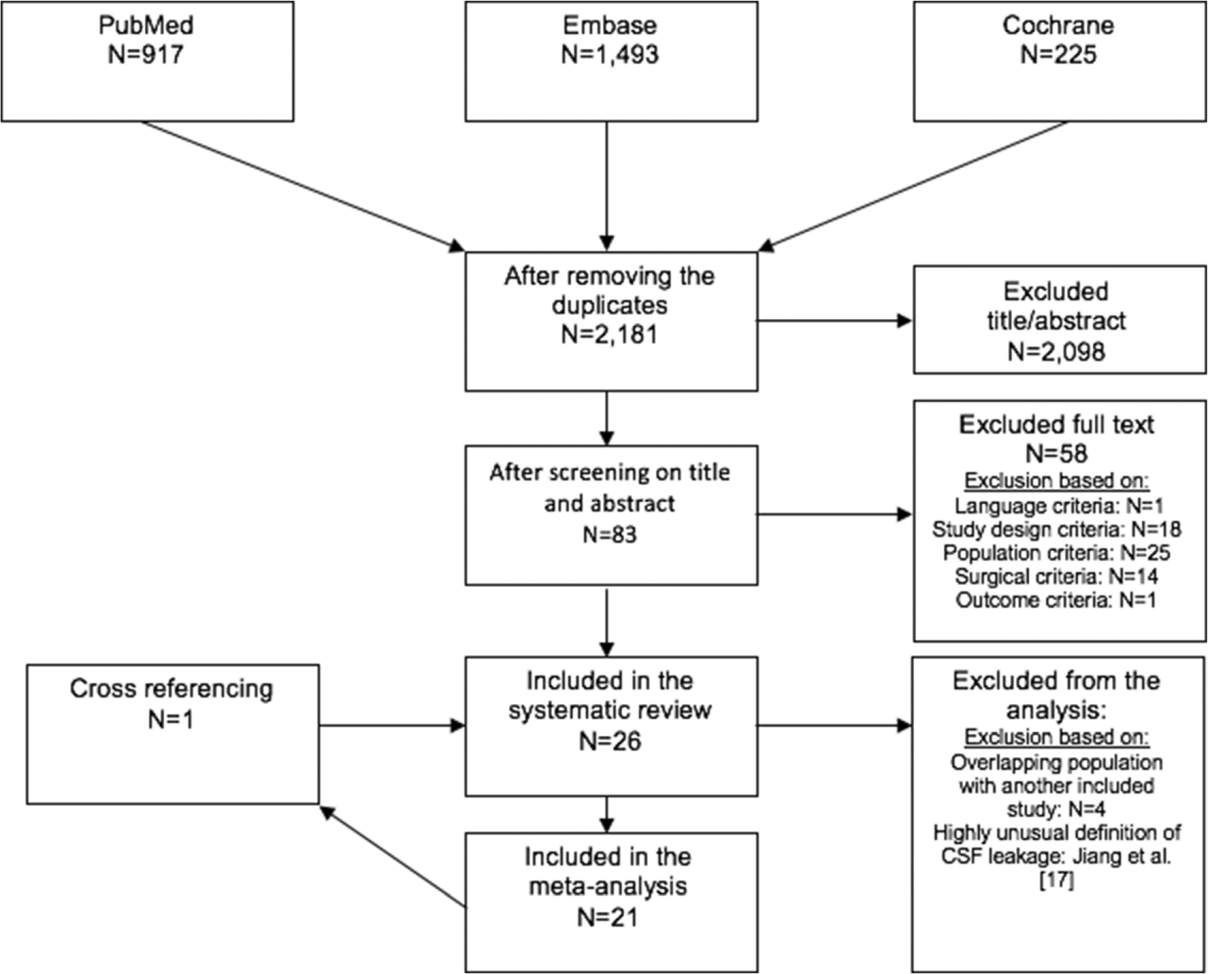
Flowchart of study selection

The database search yielded 2123 articles of which 26 were included in this systematic review (Fig. 1). Twenty-one articles were included in the meta-analysis, as four articles had to be excluded because of overlapping study populations (the article discussing the largest sample size was included) [4, 8, 21, 33]. Additionally, the study of Jiang et al. [17] was excluded from the meta-analysis, because they unconventionally diagnosed CSF leak when “drainage from the drainage catheter was clear and transparent” in their patient population in which placement of a low-vacuum suction wound drain was part of the surgical protocol.

A total of 2929 patients were included, who underwent a total of 3034 intradural cranial surgeries, as some patients had more than one surgery. Table 1 provides an overview of study characteristics.

**Table 1:** Overview of included studies

Author	Study design	Surgery type	Definition of CSF leakage	Population(*N*)	Surgeries(*N*)	Age(yrs), mean	Age(yrs), range	CSF leakageincidence (%)	Studyquality
Cochrane et al. 1994^a^ [4]	CCS	Posterior fossa tumor		91	91	7.3	0.20–16	4.4	Poor
Culley et al. 1994 [5]	CCS	Posterior fossa tumor		117	117		0.3–16	19.7	Poor
Muszynski et al. 1994 [26]	CCS	Posterior fossa tumor	CSF visibly dripping from the surgical wound	50	50		2–13	14.0	Good
Parizek et al. 1998 [29]	CCS	Posterior fossa tumor		454	439			4.6	Poor
Gnanalingham et al. 2002 [7]	CCS	Posterior fossa tumor		110	110	5.8	0.2–15	15.5	Fair
Gnanalingham et al. 2003^b^ [8]	CCS	Posterior fossa tumor		84	84	5.8	0.5–16	10.7	Fair
Bognar et al. 2003 [2]	CCS	Posterior fossa tumor		180	180	7.4	0.3–16	7.2	Poor
Steinbok et al. 2007 [35]	CCS	Posterior fossa tumor	CSF leak through the skin	154	174			10.3	Good
Gopalakrishnan et al. 2012 [9]	CCS	Posterior fossa tumor		84	84	8.0	1.5–18	2.4	Poor
Panigrahi et al. 2012 [28]	RCT	Posterior fossa tumor		14	14	8.1	2–15	0.0	Poor
Hale et al. 2019 [11]	CCS	Posterior fossa tumor		186	186	7.6	3.4–12	8.1	Fair
Kushel et al. 2019 [20]	CCS	Posterior fossa tumor		211	211			5.2	Fair
Houdemont et al. 2011 [15]	CCS	Craniotomy for tumor		99	117	7.4		6.8	Poor
Lassen et al. 2011^c^ [21]	CCS	Craniotomy for tumor	All CSF leaks andpseudomeningocelesrequiring surgical intervention	211	273	8.5	0–18	7.3	Good
Hosainey et al. 2014 [14]	CCS	Craniotomy for tumor	All CSF leaks and pseudomeningocelesrequiring surgical intervention	302	381	8.6	0–18	6.3	Good
Krieger et al. 1999 [19]	CCS	Chiari decompression		31	31		0.5–18	9.7	Fair
Parker et al. 2011[30]	CCS	Chiari decompression		114	114	8.6		5.3	Fair
Hidalgo et al. 2018 [12]	CCS	Chiari decompression		105	105	10.0		0.0	Fair
Jiang et al. 2018^d^ [17]	RCT	Chiari decompression		42	42	14.0	10–18	38.1	Fair
Zhou et al. 2014 [38]	CCS	Cranial surgery		163	160	10.2		1.3	Poor
Roth et al. 2018 [32]	CCS	Craniotomy		157	163		0.3–18	0.6	Fair
Soleman et al. 2019^e^ [33]	CCS	Interhemispheric approachfor various intracranial pathologies		26	28	10.1	2–17	0.0	Fair
Vedantam et al. 2017 [37]	CCS	Craniotomy for epilepsy		280	280		4–13	0.4	Fair
Srinivasan et al. 1999 [34]	CCS	Bifrontal olfactory nerve-sparing craniotomy		14	14	8.6	2–18	7.1	Poor
Levy et al. 2003 [23]	CCS	Microsurgical keyhole approach for middlefossa arachnoid cyst fenestration		50	50	5.7	0.1–17	6.0	Fair
Dlouhy et al. 2015 [6]	CCS	Supraorbital eyebrow craniotomy		54	54	9.6	1.5–16	0.0	Fair
Total^f^				2929	3034				

^a^Excluded from meta-analysis because of overlap with Steinbok et al. 2007

^b^Excluded from meta-analysis because of overlap with Gnanalingham et al. 2002

^c^Excluded from meta-analysis because of overlap with Hosainey et al. 2014

^d^Excluded from meta-analysis because of overestimation of CSF leakage resulting from wound drainage and inclusion of clear production in the drainage system as CSF leakage

^e^Excluded from meta-analysis because of overlap with Roth et al. 2018

^f^Studies included in the meta-analysis only

*N* number, *CSF* cerebrospinal fluid, *yrs* years, *CCS* consecutive case series, *RCT* randomized controlled trial

Most included articles report retrospective consecutive case series. One study was a randomized controlled trial, in which patients were randomized for a crescent incision versus a Y-shaped incision of the dura [28]. Ten studies were of poor quality, based on unclear description of the surgical procedure and poor definition of the outcome measure CSF leakage and either insufficient reporting of the follow up duration or lack of description of statistical methods. Twelve studies were of fair quality, again largely based on a lack of adequate definition of the outcome measures and inadequate reporting of statistical methods. Four studies were of good quality; these studies all provide a clear definition of the outcome measure CSF leakage. A detailed description of the quality assessment is presented in Supplementary Information 1.

### Primary outcome measure

**Fig. 2:**
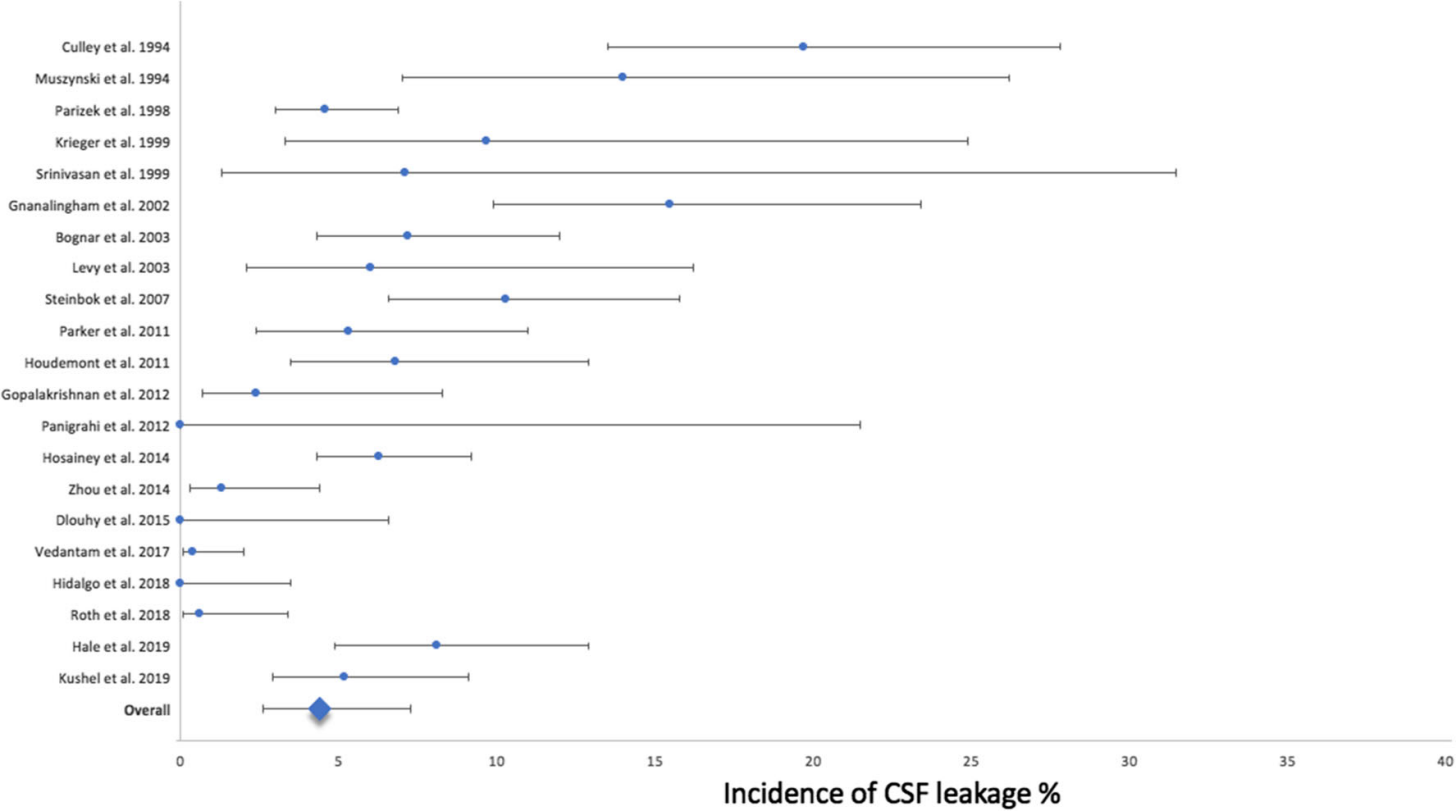
Forest plot incidence of CSF leakage

The overall incidence of CSF leakage was 4.4% (95% CI 2.6 to 7.3%) (Fig. 2).

Subgroup analyses for type of surgery could only be performed for Chiari decompression (with dural opening) and posterior fossa tumor surgery, as only these indications were investigated in sufficient studies. CSF leakage rates in these subgroups were 3.4% (95% CI 1.3 to 8.7%) after Chiari decompression, and 8.0% (95% CI 5.2–12.0%) after posterior fossa tumor surgery. All analyses showed substantial heterogeneity. An overview of outcomes for the primary outcome measure and subgroup analyses can be found in Table 2.

**Table 2:** Incidence of CSF leakage based on generalized linear mixed model

Outcome	Incidence (%)	Lower bound (%)	Upper bound (%)	Std Error	I^2^	Studies (N)	Surgeries (N)
Overall	4.4	2.6	7.3	1.1	93.6	21	3034
Posterior fossa tumor resection	8.0	5.2	12.0	1.7	87.8	10	1545
Chiari decompression	3.4	1.3	8.7	1.7	58.5	3	250

*CSF* cerebrospinal fluid

### Secondary outcome measures

The highest percentage of CSF leakage was found in patients undergoing craniectomy (10.3%, 95% CI 4.3% to 22.7%), with an OR of 4.7 (95% CI 1.7 to 13.4) compared to craniotomy (2.4%, 95% CI 1.0% to 5.4%). A CSF leakage rate of 6.4% (95% CI 4.1 to 10.0%) was found for infratentorial surgery in contrast to 1.2% (95% CI 0.4 to 3.7%) for supratentorial surgery (OR 5.9, 95% CI 1.7 to 20.6).

In patients with a duraplasty for dural closure, the incidence of CSF leakage was 5.3% whereas patients without a duraplasty had a significantly higher incidence of 11.8% (OR 0.4, 95% CI 0.2 to 0.9).

In studies in which watertight closure was attempted in all cases, the CSF leakage rate was 2**.**3% as compared to 6.4% patients in studies in which watertight closure was not attempted in all cases (OR 0.3 95% CI 0.1 to 2.3). An overview of the secondary outcome measures is presented in Table 3.

**Table 3:** Overview secondary outcome measures

Outcome	Odds ratio	Lower bound	Upper bound	*P* value	Studies (*N*)	Surgeries (*N)*
Craniectomy vs. craniotomy	4.7	1.7	13.4	0.00*	15	1917
Infratentorial vs. supratentorial	5.9	1.7	20.6	0.01*	18	2373
Duraplasty vs. no duraplasty	0.4	0.2	0.9	0.03*	5	727
Watertight closure in all cases vs. watertight closure not in all cases	0.3	0.1	2.3	0.27	10	1415

*CSF* Cerebrospinal fluid

*Significant

### Sensitivity analysis

Separate analyses were performed: (1) for studies of high quality only, (2) for studies of > 50 patients only, and (3) including the study of Jiang et al. The overall CSF leakage rate in studies of good quality [14, 26, 35] is 7.4% (95% CI 4.6 to 11.6%). For studies of more than 50 patients [2, 5, 6, 9, 11, 12, 14, 15, 17, 20, 29, 30, 32, 37], the CSF leakage rate was 3.8% (95% CI 2.0 to 7.3%). The meta-analysis including the study of Jiang et al. [17] results in an overall CSF leakage rate of 4.8% (95% CI 2.7 to 8.3%). An overview of outcomes for the sensitivity analyses can be found in Supplementary Information 2.

## Discussion

This meta-analysis shows that the overall incidence of CSF leakage after intradural cranial surgery in the pediatric population is 4.4%. Infratentorial as opposed to supratentorial surgery, and craniectomy as opposed to craniotomy are significant risk factors for CSF leakage (OR 5.9 and 4.7, respectively). These results underline the relevance of CSF leakage in clinical practice. In the pediatric population, specifically, the burden of additional treatment that may be required for CSF leakage or related complications is substantial. In studies reporting data on treatment of CSF leakage, a total of 37 out of 114 patients with a CSF leak were treated with a ventriculoperitoneal shunt [2, 5, 7, 9, 14, 19, 20, 23, 34, 35].

There is a wide range of reported CSF leakage rates (between 0.0 and 38.0%) [6, 12, 17, 28]. This may have several reasons. First, there is a large variability in the definition of CSF leakage. Moreover, only four out of 26 studies actually described their definition of CSF leakage. Secondly, the wide incidence range may be due to the different types of surgery included across studies (i.e., supra orbital eyebrow craniotomy, epilepsy surgery, posterior fossa tumor surgery).

No separate analyses could be performed per type of surgery for all these categories, nor for the risk factors like age, immune status, previous chemotherapy or previous radiotherapy, CSF diversion surgery, preoperative hydrocephalus, ventricular opening, and sealant use as there was insufficient data or only data on study level available from the included literature.

Our meta-analysis shows that the proportion of CSF leakage is the highest in the subgroup of patients undergoing craniectomy (10.3%). This difference may be explained by the lack of extra counter pressure that is otherwise provided by the replaced bone flap [7]. Replacement of the bone flap decreases the continuous short increase and decrease in dural stress caused by the triphasic pulsations of cerebrospinal fluid [3]. Furthermore, the bone flap may reduce the dead space which is created after detachment of the muscles in the suboccipital region and support their reattachment to the replaced bone flap, so that collection of CSF in this space is limited and pseudomeningocele is prevented [7].

This meta-analysis finds a CSF leakage rate of 3.4% after Chiari decompression surgery. The relatively low leakage rate in this population is surprising considering the abovementioned surgical risk factors (infratentorial surgery and craniectomy) as this population essentially represents a combination of these two items.

On the contrary, a high leakage rate in posterior fossa tumor surgery (8.0%) is found. This type of surgery may be prone for leakage because pediatric brain tumors frequently reside in the fourth ventricle, requiring opening of the telovelar membrane and leaving a wide-open ventricle. Furthermore, postoperative hydrocephalus may contribute to the increased incidence of CSF leakage in this population [11].

The effect of watertight closure was not significant in this study. However the effect in this analysis may be limited because it was only possible to compare studies in which all cases were closed in watertight fashion to those in which not all cases were closed with this aim (the dura was left open in all cases in one study [19], in other studies 10–89% [26, 29, 32] of cases were not closed in a watertight manner).

CSF leakage was significantly less frequent in patients in whom a duraplasty was performed (OR 0.4). This may reflect that when careful attention is paid to optimal closure of the dura with or without augmentation such as duraplasty or sealants, the risk of CSF leakage is reduced. No distinction has been made in this study between autologous or synthetic material. A study by Hale et al. (2020) indicates that graft dural closure may furthermore be protective against hydrocephalus and wound infection in patients undergoing posterior fossa tumor surgery [11].

Compared to adults, the incidence of CSF leakage found in children is considerably lower, which is contrary to our expectations considering the high number of craniectomies and infratentorial surgeries included. A recent meta-analysis has found that the rate of CSF leakage in adults is 8% [18]. As is the case in pediatric literature, the definition of CSF leakage reported in studies on adults is not uniform either. This may explain the discrepancy between the incidence of CSF leakage in both populations. Another factor may be that the meta-analysis on adults includes studies in which sealants use was compared, this patient population may, therefore, be one which is more prone to CSF leakage, considering a substantial number of studies selected patients based on intraoperative CSF leakage. Moreover, this may be a result of increased flexibility of the tissues in children compared to adults allowing for better surgical closure of the dura and skin layers.

This meta-analysis is subject to several limitations. Most importantly, the studies included are heterogenous in their definitions of the outcome measure, population, and follow-up duration. The majority of studies included in this meta-analysis do not clearly define the outcome measure CSF leakage. Those that do, use a variety of definitions, for example, being “CSF leak through the skin” [35] and “all CSF leaks requiring surgical intervention” [14]. This obviously results in differences in outcome, as is reflected by the *I*^2^-values found in the meta-analyses. It was not possible to adopt a specific definition of CSF leakage for this meta-analysis, as too few publications mention this. One study has been excluded because it included clear fluid in a low-vacuum suctioning wound drainage system as CSF leakage, resulting in an outstandingly high CSF leakage rate of 38.0% [17]. In a sensitivity analysis including this publication, we found an overall CSF leakage rate of 4.8% (4.4% without), indicating this study has no clinically meaningful influence on the overall outcome.

Secondly, the risk factor analyses for duraplasty use and watertight closure were based on a limited number of studies. Therefore, caution should be applied in generalizing these results.

Thirdly, we did not exclude patients with subdural-to-extracranial implants, such as subdural grid electrodes, which may influence CSF leakage, but the total influence of this population on the overall results is expected to be minimal.

Fourth, the results of the risk factor analysis are potentially influenced by confounding. This is inherent to the design of the included publications and the fact that obtained data do not allow correction for potential bias. Future research should further investigate potential risk factors in a multivariate analysis.

Lastly, quality assessment identified only 3 “good quality” studies out of the 26 included in the meta-analysis, compromising quality for the reported outcome measure. The sensitivity analysis shows a higher incidence of CSF leakage in studies of good quality, 7.4% vs. 4.4% found in all studies which may indicate that the CSF leakage rate in this study may be an underestimation of the true CSF leakage rate.

Despite these limitations, this meta-analysis provides a representative overview of the CSF leakage rate and associated risk factors reported in the current body of literature. Moreover, it emphasizes the need for a uniform definition and future studies evaluating CSF leakage and preventative strategies in the pediatric population. CSF leakage may include both incisional leakage and pseudomeningocele (PMC). Incisional CSF leakage is defined as leakage of CSF through the skin, whereas a PMC is an extradural collection of CSF under the skin [24]. Although PMC in the absence of incisional CSF leakage can cause symptoms such as, intracranial hypotension, aseptic meningitis, pain, and psychological distress, the condition is often self-limiting [24, 36]. Describing and quantifying symptomatic PMC can be difficult because the diagnosis is subjective in contrast to incisional CSF leakage. Therefore, it should be considered a separate entity.

## Conclusions

The overall CSF leakage rate after intradural cranial surgery in the pediatric population is 4.4%. The highest leakage rate is found in patients undergoing a craniectomy. Infratentorial surgery is also associated with higher incidence of CSF leakage, whereas the use of a duraplasty is negatively associated with CSF leak. We emphasize the need for a uniform and clinically meaningful definition of CSF leakage, suggesting “leakage of CSF through the skin.”

### Supplementary Information

ESM 1 (DOCX 19 kb)

ESM 2 (DOCX 14 kb)

## Pubmed search

(“Craniotomy”[Mesh] OR Craniotom*[Title/Abstract] OR Craniectom*[Title/Abstract] OR cranial surgery [Title/Abstract] OR tumor resect*[Title/Abstract] OR tumour resect*[Title/Abstract] OR neurosurgery*[Title/Abstract])

AND

(“Cerebrospinal Fluid Leak”[Mesh:NoExp] OR Cerebrospinal Fluid Leak*[Title/Abstract] OR CSF leak*[Title/Abstract] OR pseudomeningocele [Title/Abstract] OR incisional leak*[Title/Abstract] OR “Meningitis”[Mesh] OR meningitis [Title/Abstract] OR “Surgical Wound Infection”[Mesh] OR Surgical Wound Infection*[Title/Abstract] OR wound infection*[Title/Abstract] OR wound leak*[Title/Abstract] OR surgical site infection*[Title/Abstract])

AND

(Infan*[Title/Abstract] OR toddler*[tiab] OR minor [tiab] OR minors*[tiab] OR boy [tiab] OR boys[tiab] OR girl[tiab] OR girls[tiab] OR kid[tiab] OR kids[tiab] OR child[tiab] OR children*[tiab] OR adolescen*[tiab] OR juvenil*[tiab] OR youth*[tiab] OR teen*[tiab] OR pediatrics[MESH] OR pediatri*[tiab] OR paediatri*[tiab] OR youth[tiab] OR youths[tiab] OR teen[tiab] OR teens[tiab] OR teenager[tiab] OR youngster*[tiab] OR child[MeSH])

## Embase search

(‘craniotomy’/exp OR ‘craniotom*’:ab,ti OR ‘craniectom*’:ab,ti OR ‘cranial surgery’:ab,ti OR ‘tumor resect*’:ab,ti OR ‘tumour resect*’:ab,ti OR neurosurgery*:ab,ti)

AND

(‘liquorrhea’/exp/mj OR ‘cerebrospinal fluid leak*’:ab,ti OR ‘csf leak*’:ab,ti OR pseudomeningocele:ab,ti OR ‘incisional leak*’:ab,ti OR ‘meningitis’/exp OR meningitis:ab,ti OR ‘surgical infection’/exp OR ‘surgical wound infection*’:ab,ti OR ‘wound infection*’:ab,ti OR ‘wound leak*:ab,ti’ OR ‘surgical site infection*’:ab,ti)

AND

(infan*:ab,ti OR toddler*:ab,ti OR minor:ab,ti OR minors*:ab,ti OR boy:ab,ti OR boys:ab,ti OR girl:ab,ti OR girls:ab,ti OR kid:ab,ti OR kids:ab,ti OR child:ab,ti OR children*:ab,ti OR adolescen*:ab,ti OR juvenil*:ab,ti OR youth*:ab,ti OR teen*:ab,ti OR 'pediatrics'/exp OR pediatri*:ab,ti OR paediatri*:ab,ti OR youth:ab,ti OR youths:ab,ti OR teen:ab,ti OR teens:ab,ti OR teenager:ab,ti OR youngster*:ab,ti OR 'child'/exp)

AND

[embase]/lim

## Cochrane search

MeSH descriptor: [Craniotomy] explode all trees OR craniotom* OR craniectom* OR cranial surgery OR tumor resect* OR tumour resect* OR neurosurger*

AND

MeSH descriptor: [Cerebrospinal Fluid Leak] explode all trees OR MeSH descriptor: [Meninges] explode all trees OR MeSH descriptor: [Surgical Wound Infection] explode all trees OR Cerebrospinal Fluid Leak OR CSF leak* OR pseudomeningocele OR incisional leak*OR OR meningitis OR Surgical Wound Infection* OR wound infection* OR wound leak*OR surgical site infection*

AND

MeSH descriptor: [Pediatrics] explode all trees OR MeSH descriptor: [Child] explode all trees OR Infan* OR toddler* OR minor OR minors* OR boy OR boys OR girl OR girls OR kid OR kids OR child OR children* OR adolescen* OR juvenil* OR youth* OR teen* OR pediatri* OR paediatri*OR youth OR youths OR teen OR teens OR teenager OR youngster*
