# Supplementary material for: Cerebrospinal fluid leakage after cranial surgery in the pediatric population—a systematic review and meta-analysis
Source: Childs Nerv Syst. 2021 Feb 4;37(5):1439–47. doi: 10.1007/s00381-021-05036-8 (PMC8084768; doi:10.1007/s00381-021-05036-8)
Supplement: Supplementary file 1 — (DOCX 19 kb) [file 381_2021_5036_MOESM1_ESM.docx]

**Cerebrospinal fluid leakage after cranial surgery in the pediatric population – A systematic review and meta-analysis**

Child’s Nervous System

*Emma M.H. Slot, MD^1^, Kirsten M. van Baarsen, MD, PhD^2^, Eelco W. Hoving, MD, PhD^1,2^, Nicolaas P.A. Zuithoff, PhD^3^, Tristan P.C van Doormaal, MD, PhD^1,4^*

^1^Department of Neurology and Neurosurgery, University Medical Center Utrecht, Utrecht, The Netherlands

^2^Department of Neuro-oncology, Princess Máxima Center for Pediatric Oncology, Utrecht, The Netherlands

^3^Julius Center for Health Sciences and Primary Care, University Medical Center Utrecht, Utrecht, The Netherlands

^4^Department of Neurosurgery, University Hospital Zürich, Zürich, Switzerland

Corresponding author:

E.M.H. Slot, MD

Department of Neurology and Neurosurgery

University Medical Center Utrecht, Str. 4.123

Heidelberglaan 100

3584 CX Utrecht, The Netherlands

e.m.h.slot-4@umcutrecht.nl

**Supplementary Information 1.** Overview of quality assessment

| **Study** | **Question** | | | | | | | | | **Quality rating** |
| --- | --- | --- | --- | --- | --- | --- | --- | --- | --- | --- |
|  | Was the study question or objective clearly stated? | Was the study population clearly and fully described, including a case definition? | Were the cases consecutive? | Were the subjects comparable? | Was the intervention (surgery) clearly described? | Were the outcome measures (CSF leakage) clearly defined, valid, reliable, and implemented consistently across all study participants? | Was the length of follow-up adequate  (one month or longer)? | Were the statistical methods well-described (statistical test mentioned)? | Were the results well-described (percentage of CSF leakage or number of CSF leakage mentioned)? |  |
| **Cochrane 1994^a^** | Yes | Yes | Yes | Yes | No | No | NR | NR | Yes | **Poor** |
| **Bognar 2003** | Yes | Yes | Yes | Yes | No | No | Yes | Yes | Yes | **Poor** |
| **Zhou 2014** | Yes | Yes | Yes | Yes | No | No | Yes | No | Yes | **Poor** |
| **Culley 1994** | Yes | Yes | Yes | Yes | No | No | NR | Yes | Yes | **Poor** |
| **Gopalakrishnan 2012** | Yes | Yes | Yes | Yes | No | No | NR | Yes | Yes | **Poor** |
| **Houdemont 2011** | Yes | Yes | Yes | Yes | No | No | NR | Yes | Yes | **Poor** |
| **Parizek 1998** | Yes | Yes | Yes | Yes | Yes | No | NR | NR | Yes | **Poor** |
| **Srinivasan 1999** | No | Yes | Yes | Yes | Yes | No | Yes | NA | Yes | **Poor** |
| **Panigrahi 2012** | Yes | Yes | CD | Yes | Yes | No | NR | NA | Yes | **Poor** |
| **Hale 2019** | Yes | Yes | Yes | Yes | No | No | NR | Yes | Yes | **Poor** |
| **Krieger 1999** | Yes | Yes | Yes | Yes | Yes | No | Yes | NR | Yes | **Fair** |
| **Dlouhy 2015** | Yes | Yes | Yes | Yes | Yes | No | Yes | NR | Yes | **Fair** |
| **Hidalgo 2018** | Yes | Yes | Yes | Yes | Yes | No | Yes | NR | Yes | **Fair** |
| **Vedantam 2017** | Yes | Yes | CD | Yes | Yes | No | Yes | Yes | Yes | **Fair** |
| **Gnanaligham 2003^a^** | Yes | Yes | Yes | Yes | Yes | No | Yes | Yes | Yes | **Fair** |
| **Gnanaligham 2002** | Yes | Yes | Yes | Yes | Yes | No | Yes | Yes | Yes | **Fair** |
| **Kushel 2019** | Yes | Yes | Yes | Yes | Yes | No | Yes | Yes | Yes | **Fair** |
| **Levy 2003** | Yes | Yes | Yes | Yes | Yes | No | Yes | NR | Yes | **Fair** |
| **Parker 2011** | Yes | Yes | Yes | Yes | Yes | No | Yes | Yes | Yes | **Fair** |
| **Soleman 2019^a^** | Yes | Yes | Yes | Yes | Yes | No | Yes | Yes | Yes | **Fair** |
| **Jiang 2018^b^** | Yes | Yes | Yes | Yes | Yes | No | Yes | Yes | Yes | **Fair** |
| **Roth 2018** | Yes | Yes | Yes | Yes | Yes | No | Yes | Yes | Yes | **Fair** |
| **Lassen 2011^a^** | Yes | Yes | Yes | Yes | Yes | Yes | Yes | Yes | Yes | **Good** |
| **Muszynski 1994** | Yes | Yes | Yes | Yes | Yes | Yes | Yes | Yes | Yes | **Good** |
| **Hosainey 2014** | Yes | Yes | Yes | Yes | Yes | Yes | Yes | Yes | Yes | **Good** |
| **Steinbok 2007** | Yes | Yes | Yes | Yes | Yes | Yes | NR | Yes | Yes | **Good** |

| **Legend** | |
| --- | --- |
| ^a^ | not included in meta-analysis because of overlap with another study included in the meta-analysis. |
| ^b^ | not included in meta-analysis because of overestimation of CSF leakage because overestimation of CSF leakage resulting from wound drainage and inclusion of clear production in the drainage system as CSF leakage |
| CD | Cannot determine |
| NA | Not applicable |
| NR | Not reported |
| Yes | Low risk of bias |
| CD/NA/NR | Unclear risk of bias |
| No | High risk of bias |
