# Supplementary material for: Cerebrospinal fluid leakage after cranial surgery in the pediatric population—a systematic review and meta-analysis
Source: Childs Nerv Syst. 2021 Feb 4;37(5):1439–47. doi: 10.1007/s00381-021-05036-8 (PMC8084768; doi:10.1007/s00381-021-05036-8)
Supplement: Supplementary file 2 — (DOCX 14 kb) [file 381_2021_5036_MOESM2_ESM.docx]

**Cerebrospinal fluid leakage after cranial surgery in the pediatric population – A systematic review and meta-analysis**

Child’s Nervous System

*Emma M.H. Slot, MD^1^, Kirsten M. van Baarsen, MD, PhD^2^, Eelco W. Hoving, MD, PhD^1,2^, Nicolaas P.A. Zuithoff, PhD^3^, Tristan P.C van Doormaal, MD, PhD^1,4^*

^1^Department of Neurology and Neurosurgery, University Medical Center Utrecht, Utrecht, The Netherlands

^2^Department of Neuro-oncology, Princess Máxima Center for Pediatric Oncology, Utrecht, The Netherlands

^3^Julius Center for Health Sciences and Primary Care, University Medical Center Utrecht, Utrecht, The Netherlands

^4^Department of Neurosurgery, University Hospital Zürich, Zürich, Switzerland

Corresponding author:

E.M.H. Slot, MD

Department of Neurology and Neurosurgery

University Medical Center Utrecht, Str. 4.123

Heidelberglaan 100

3584 CX Utrecht, The Netherlands

e.m.h.slot-4@umcutrecht.nl

**Supplementary Information 2.** Overview of sensitivity analyses.

| Analysis | Incidence (%) | Lower bound (%) | Upper bound (%) | Std Error (%) | I^2^ | Studies (N) | Surgeries (N) |
| --- | --- | --- | --- | --- | --- | --- | --- |
| High quality studies | 7.4 | 4.6 | 11.6 | 1.2 | 62.4 | 5 | 806 |
| Studies > 50 patients | 3.8 | 2.0 | 7.3 | 1.2 | 92.4 | 16 | 2,875 |
| Including Jiang et al. | 4.8 | 2.7 | 8.3 | 1.3 | 94.3 | 22 | 3,076 |
